# The Value of Electroencephalogram (EEG) Findings in the Evaluation and Treatment Management of Pediatric Acute Liver Failure

**DOI:** 10.7759/cureus.54300

**Published:** 2024-02-16

**Authors:** Bilge Özgör, Sukru Gungor, Merve Aladağ, Fatma İ Varol, Mahmut Aslan, Sezai Yilmaz, Serdal Gungor

**Affiliations:** 1 Pediatric Neurology, İnönü University, Malatya, TUR; 2 Pediatric Gastroenterology, İnönü University, Malatya, TUR; 3 Pediatric Medicine, Nurdağı State Hospital, Gaziantep, TUR; 4 Pediatric Neurology, Mersin City Hospital, Mersin, TUR; 5 General Surgery, İnönü University, Malatya, TUR

**Keywords:** management, prognosis, treatment, eeg, pediatric acute liver failure

## Abstract

Background

Pediatric acute liver failure (PALF) is still life-threatening and requires urgent care. The presence of encephalopathy is a clinical diagnosis, but it is more difficult to diagnose in children than in adults, and an electroencephalogram (EEG) can be invaluable. The role of EEG in managing the treatment of patients with PALF, other than the identification of encephalopathy, is unknown. This study aimed to investigate patients' EEGs, which may guide in choosing the most appropriate treatment in encephalopathy children. A further aim was to investigate a new score method, based on the laboratory results, which might indicate the presence of encephalopathy in cases with PALF.

Methods

Medical data of 33 PALF patients followed in our clinic were reviewed retrospectively. This study included 33 patients, whose EEG recording was taken on the first day of supportive treatment due to liver failure in the pediatric intensive care unit (PICU). The EEG findings were categorized into three classes: normal, epileptic and non-epileptic paroxysmal, and background encephalopathic patterns including widespread slowing and voltage suppression.

Result

This retrospective study included 13 male and 20 female patients with a mean age at presentation of 4.82±4.81 months whose EEG was performed on the first day of supportive therapy for liver failure in the PICU. The EEG findings were categorized into three groups: normal, epileptic and non-epileptic paroxysms, and encephalopathic patterns including diffuse background slowing and voltage suppression. Comparing EEG findings and treatments, we found that the normal EEG group responded well to liver-supporting therapy and the rate of plasmapheresis treatment was significantly higher in the diffuse slowing group. Patients with diffuse slowing of the EEG were 9.6 times more likely to receive plasmapheresis. We found that above a cut-off of ≥7.5 for the TAI (total bilirubin, albumin, and international normalized ratio (INR)) score used in our study, the risk of developing encephalopathy increased 14.4-fold.

Conclusions

In PALF, EEG findings can provide findings that will help clinicians in determining treatment selection and prognosis, as well as detecting epileptic focus and encephalopathy. The TAI score can be used to assess the risk of encephalopathy in cases of PALF, when it is challenging to identify encephalopathy or when an EEG is not possible.

## Introduction

Pediatric acute liver failure (PALF) is a life-threatening and rapidly progressive clinical syndrome that has been caused by medications, toxins, immune dysregulation diseases, inherited metabolic diseases, infectious diseases, and idiopathic etiologies. The frequency of PALF is not known, but PALF's estimated incidence is 10% of pediatric liver transplants. When the clinician encounters a PALF child, its management requires a multidisciplinary team and the evaluation of the fourth issue: "Is it treatable, can the child live without their liver or will they deteriorate, is liver transplantation necessary for survival, and are the associated morbidities reversible?" [1]. An intensive care setting is necessary day and night to assess the evidence of changing mental status or hepatic encephalopathy (HE), increased respiratory effort, changing heart rate or changes in blood pressure that might be signs of infection, increasing cerebral edema, or electrolyte imbalance for a PALF patient, and these parameters must be closely monitored.

Many expert clinicians have used distinct models for estimating predicted death such as the King's College Hospital criteria, the Clichy score, the Model for End-Stage Liver Disease (MELD) score, and the Pediatric End-Stage Liver Disease (PELD) score. All those tests including PELD are weakly associated with outcomes in children with acute liver failure although PELD is the more useful score for chronic liver failure patients because it includes growth evaluation parameters [2]. The Liver Injury Unit score has been developed specifically for PALF by the Pediatric Acute Liver Failure Study Group that has been including factors for peak total bilirubin, prothrombin time (PT) or international normalized ratio (INR), and ammonia. Although the Liver Injury Unit score is somewhat more useful, it is not sufficient to make critical decisions about liver transplantation [3]. The Child-Pugh scoring system (also known as the Child-Pugh-Turcotte score) is a predictor of mortality in patients with chronic liver failure. This system was first described by Child and Turcotte in 1964 as a guide to the selection of patients who would benefit from elective portal decompression surgery and divided patients into three categories: good liver function, moderately impaired liver function, and severely impaired liver function. The original scoring system used to have three clinical criteria (ascites, neurological impairment, and clinical nutrition status) and two laboratory criteria (serum bilirubin, serum albumin) for grading patients [4]. The scoring system was modified by Pugh et al. who used albumin, PT, and INR instead of the clinical nutritional status. They also defined variable scores according to the severity of liver failure [5]. Although PELD scoring or the Child-Pugh-Turcotte score is a predictive method for children with chronic liver failure, an evidence-based method has not yet been developed for PALF [6]. Furthermore, these scoring methods inform about mortality but do not inform about treatment management.

However, the most reliable sign of progression of PALF is the presence of encephalopathy, and it is particularly challenging to diagnose encephalopathy in children [6]. The diagnosis of encephalopathy is made clinically by anamnesis, consciousness, and detailed neurological examination. Electroencephalography (EEG) interpreted by specialized physicians is informative in recognizing encephalopathy. EEG is a sensitive and rapid method to identify cerebral disorders by recording brain electrical signals. EEG informs the intensity and patterns of brain electrical activity, brain waves during sleep-wake, epilepsy, anesthesia, or coma. Regarding the level of activity in the cerebral cortex, the form of the waves (alfa, beta, delta, theta, and gamma) shows very different patterns in sleep, wakefulness, and coma. The main advantages of EEG are its high temporal resolution, non-invasiveness, and painlessness method [7]. Although an EEG shows postsynaptic electrical activity, it cannot give information about the inside electrical activity in the neuron. Also, electrical activity in deep tissues cannot be screened on the scalp electrode recordings. Numerous artifacts (inappropriate preparation of the patient's scalp, loss of head integrity, motor movements of the patient and/or technician, electrical artifacts of the devices and/or system) that occur during EEG acquisition can lead to inadequate or biased recording interpretation. EEG is inexpensive and non-invasive when used properly by professionals. Thus, abnormal EEG has been noted as an early diagnostic sign of brain dysfunction and guides with expert opinion in the absence of overt or minimal HE clinic [8-13].

An urgent priority in the management of children with acute liver failure is the development of prognostic markers or objective markers of mortality risk. For PALF patients without a treatable underlying cause or etiological contraindications, liver transplantation is the gold standard of care. Especially in countries where organ donation is insufficient, these patients might lose their chance of a transplant if they are added to the transplant extensive list within 48 hours of admission. The question of which patient should receive which treatment and when in PALF patients is still unclear to ensure that these lists can be drawn up efficiently. This study aimed to investigate EEG which might have been a predictive value of the outcome and guide in choosing the optimal treatment management for encephalopathic PALF children. A further aim was to investigate a new score method, based on the laboratory results, which might indicate the presence of encephalopathy in cases with PALF where an EEG was unavailable or encephalopathy is difficult to diagnose.

## Materials and methods

In our study, patients with acute hepatic failure who were admitted to the Pediatric Neurology Department of İnönü University Faculty of Medicine in Malatya, Turkey, retrospectively between 2015 and 2021 were evaluated. The number of liver transplantations is approximately 270-300 per year in all ages in our hospital. Following the International Classification of Diseases (ICD)-10, international diagnostic codes were examined in the files.

The diagnosis of acute liver failure was determined according to the diagnostic criteria of the Pediatric Acute Liver Failure Study Group as follows: (1) children (aged one month to 18 years) without evidence of chronic liver disease, (2) biochemical evidence of acute liver injury, and (3) liver-based coagulopathy (defined as PT ≥15 s or INR ≥1.5 uncorrected with vitamin K in the presence of HE or PT ≥20 s or INR ≥2.0 regardless of the presence or absence of clinical encephalopathy).

HE is classically graded by the West Haven criteria which categorizes HE from grade I to grade IV based on various clinical parameters such as alteration in the level of consciousness, intellectual function and behavior, neurological examination, and presence or absence of asterixis [5].

Inclusion criteria

Patients aged one month to 18 years who had been diagnosed with PALF, who underwent EEG recording on the first day of treatment, and who were evaluated with a neurological examination by a pediatric neurologist once a day thereafter were included in the study.

Exclusion criteria

Patients who did not meet the diagnostic criteria for PALF, those with incomplete or inadequate medical records, those diagnosed with chronic liver disease without acute liver failure findings, those who were newborns or premature at the time of acute liver failure findings, and those who did not have an EEG recording on the first day of treatment were excluded from the study.

**Figure 1:**
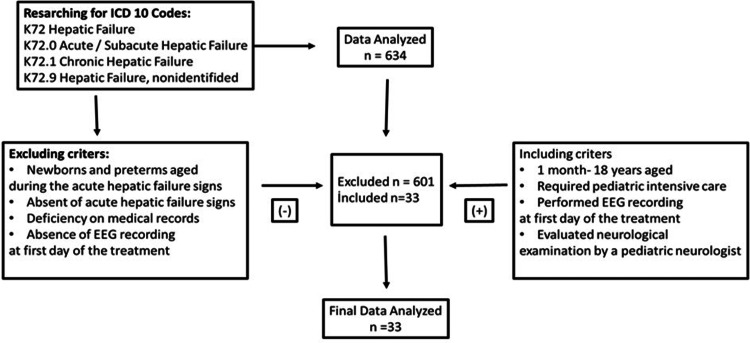
Flowchart of study inclusion and exclusion criteria

Figure 1 shows the flowchart of study inclusion and exclusion criteria.

Patients

**Figure 2:**
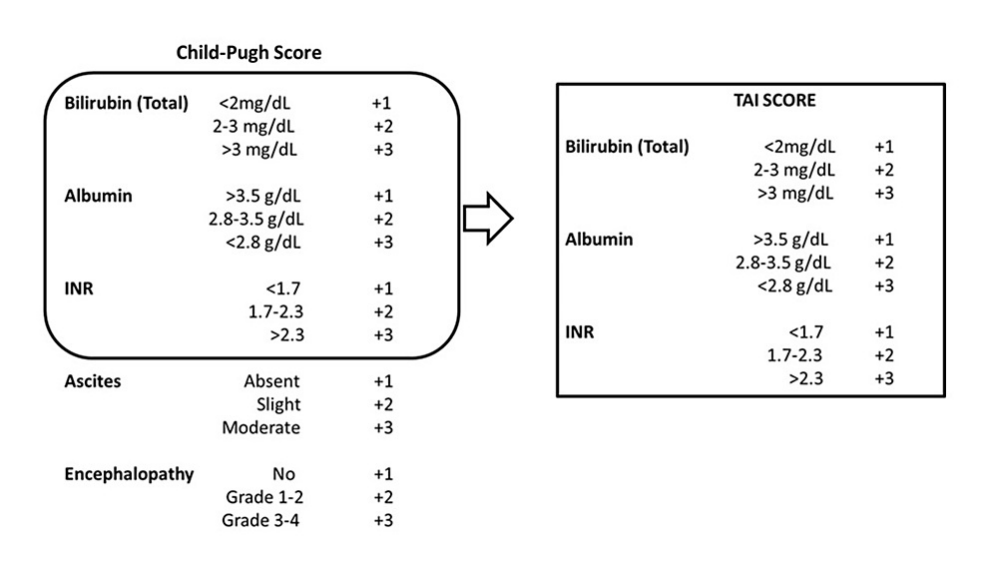
The criteria of Child-Pugh and TAI scores TAI: total bilirubin, albumin, and international normalized ratio

All the children admitted to the pediatric intensive care unit (PICU) of Turgut Özal Medical Center who met the criteria for our PALF study from January 2015 to September 2021 were collected. The demographic findings, clinical symptoms, Pediatric End-Stage Liver Disease (PELD)/Model for End-Stage Liver Disease (MELD) and Child-Pugh scores, laboratory results, HE stage and etiology, EEG, and medical and surgical treatments are listed in Table 1. The staging of PELD/MELD and Child-Pugh scores of each patient was assessed by a pediatric hepatologist according to published criteria [2,10-15] (Figure 2). The MELD scoring system has been used in four patients aged older than 12 years and the PELD scoring system in 29 patients. The clinical data were abstracted from the electronic medical records.

**Table 1:** Patients' demographic and clinical findings Statistical methods: descriptive, frequency PELD: Pediatric End-Stage Liver Disease; MELD: Model For End-Stage Liver Disease; ALT: alanine transaminase; INR: international normalized ratio; EEG: electroencephalogram

n=33	Mean±SD (min-max)
Age (years)	4.82±4.81 (0-17)
Child-Pugh score	10.48±1.87 (7-14)
PELD and MELD score	27.67±11.63 (3-54)
Hospitalization (days)	47.09±50.74 (3-250)
Ammonia (mmol/L)	254.15±256.86 (51-1428)
Albumin (mg/dL)	2.82±0.74 (1.4-4.3)
Total bilirubin (mg/dL)	17.9±15.5 (1-64)
ALT (IU)	1459.54±2153.79 (89-7273)
INR	4.26±3.13 (1.6-12.87)
Demographical findings	
Gender	n (%)
Female	20-60.6
Male	13-39.4
Consanguineous	22-66.7
Clinical findings	
Encephalopathy	23-69.7
Icter	24-72.7
Seizure	7-23.9
Ascites	7-21.2
Encephalopathy stages	
No encephalopathy	10-30.3
Stage 1 encephalopathy	8-24.2
Stage 2 encephalopathy	3-9.1
Stage 3 encephalopathy	11-33.3
Stage 4 encephalopathy	1-3
Etiology	
Idiopathic	8-24.2
Toxic hepatitis	10-30.3
Cholestasis	6-18.1
Infection	6-18.1
Wilson disease	3-9
EEG findings	
Normal	10-30
Epileptic	8-24
Diffuse slowing	12-36
Voltage suppression	3-9
Treatment	
Liver support treatment	33-100
Plasmapheresis	22-66.7
Liver transplantation	19-57.6

We have developed a new scoring system to detect the presence of encephalopathy based on laboratory parameters. It is intended to be used in patients with PALF in cases where encephalopathy cannot be detected and neurological examination does not provide sufficient information. The new scoring system we used in our study by removing the clinical parameters of the Child-Pugh scoring system includes bilirubin, INR, and albumin values. This scoring, which we called TAI (total bilirubin, albumin, and INR), was calculated for all cases (Figure 2).

Causes of PALF were classified as indeterminate, infectious, cholestasis, Wilson disease, toxic hepatitis, and others. The abstracted laboratory data included total bilirubin, alanine transaminase, INR, and ammonia recorded until PALF resolved, liver transplantation, or death. Any changes were not made in the clinical management of PALF, laboratory studies, and electrophysiological findings which were obtained according to routine clinical practice.

The clinical neurologic status and staging of HE of each patient were assessed at least daily by a pediatric neurologist. The assessment and grading of HE was classified according to published criteria for children [2]. The HE score was discussed with the attending pediatric hepatologist and decided together. Informed consent was obtained from all subjects involved in the study.

EEG

The digital EEG recordings were performed with 22 scalp electrodes (Fp1, F7, T3, T5, T7, C3, P3, O1, Fp2, F8, T4, T6, T8, C4, P4, O2, Fz, Cz, Pz, Oz, ear, and reference) and 16 channels (longitudinal montage, transverse montage, and average montage) by using the international 10-20 system of electrode placement according to the American Clinical Neurophysiology Society guidelines with EEG monitoring (Natus® NeuroWorks® EEG Software, Middleton, Wisconsin, United States) [15]. EEG was performed with non-invasive scalp electrodes made of non-polarized material (gold) and non-polarized material (gold) with a cupid-tipped electrode with a 10-mm-diameter conductive paste on the contact surface. Before each was performed, the high-pass and low-pass filter settings were verified as 1 Hz for the high-pass and 70 Hz for the low-pass digital filter value. The notch filter set at 50 Hz in Europe was used to avoid electrical artifacts caused by the electrical source. Calibration was performed to assess the accuracy of the EEG apparatus before each recording. Recordings were performed for a minimum of one hour. The length of the study was based on ongoing EEG results and the clinical status of the patient. EEG abnormalities were classified as background slowing (focal and generalized slowing, attenuation, reduced organization, or complexity), voltage suppression, interictal epileptiform abnormalities (focal spikes and/or sharp waves, generalized discharges), specific and non-epileptic patterns (periodic lateralized epileptiform discharges, suppression bursts, triphasic waves, frontal intermittent rhythmic delta activity, focal slow waves, and electrocerebral silence), and ictal abnormalities (electroclinical seizures, electrographic-only seizures). Voltage suppression may vary depending on the assembly and filters evaluated. However, voltage amplitudes of less than 20 µV were considered as voltage suppression. All EEG recordings were reported by a pediatric neurologist.

EEG findings were staged into three stages. Stages included normal, epileptic-non-epileptic patterns (such as sharps, spikes, slow waves, and fast rhythms), and encephalopathy (including diffuse slowing and voltage suppression anomalies), respectively. Epileptic EEG findings were distinct from the encephalopathy.

Neuroimaging, using cranial CT or MRI, was obtained within the first 48 hours of admission in patients in whom neurologic compromise was suspected.

Medical treatment

The use of hyperosmolar therapies (mannitol or hypertonic saline) and plasmapheresis was recorded. Anti-epileptic treatment was performed in recurrent seizures, and the first choice of drug was levetiracetam.

Ethics approval

Our study was approved by the Ethical Committee of Research of the Faculty of Medicine, İnönü University (approval number: 2021/1313).

Statistical analysis

No studies were found that predicted encephalopathy with laboratory results in the setting of acute liver failure. Therefore, using an effect size of 0.55, a power of 0.85, and a critical t value of 1.71, the number of patients was calculated to be a minimum of 27. The actual power value was 0.86. G*Power 3.1 was used to analyze the sample size.

Statistical analyses were performed using IBM SPSS Statistics for Windows, Version 20.0 (Released 2011; IBM Corp., Armonk, New York, United States). The normality of the distribution of the data was tested using visual (histogram and probability charts) and analytical methods (Kolmogorov-Smirnov and Shapiro-Wilk tests). Descriptive analyses were presented as percentile, mean, and standard deviation. Normally distributed numerical data were compared using the independent samples t-test, and non-normally distributed numerical data were compared using the Mann-Whitney U test. The chi-squared test was used to compare the frequency rates of categorical variables. Receiver operating characteristic (ROC) curve analysis was performed to determine the best cut-off points for TAI and Child-Pugh score measurements to detect encephalopathy. Logistic regression analysis was performed to evaluate whether the TAI score cut-off point determined by ROC curve analysis poses a risk for encephalopathy. A p-value of <0.05 was considered statistically significant.

## Results

Regarding ICD-10 codes as shown in Figure 1, 634 patients' electronic data had been evaluated. After the election of the files, 601 cases were excluded from the study, because of 53 patients with missing recorded data, 62 patients with chronic liver failure, and 486 patients whose EEG could not be taken before treatment for acute liver failure. Finally, 33 patients were included in our study. As a result, 19 of 33 patients had liver transplantation. Six of these patients died after transplantation. Fourteen patients were given liver support therapy. Five of the patients who received liver support treatment did not respond to the treatment and died because the donor could not be found.

The mean age of the patients was 4.82 years and the age spectrum ranged from one month to 18 years. Twenty (60.6%) of these patients were females and 13 were males. There was consanguinity between parents in 22 (66.7%) patients. Jaundice was detected in 24 patients (72.7%), encephalopathy in 23 patients (69.7%), and ascites in seven patients (21.2%) during admission. Stage 3 encephalopathy was frequently observed in patients (11 cases, 33.3%) with encephalopathy and 10 cases without encephalopathy. Toxic hepatitis was the most etiology in our study with 10 cases. The other etiologies were idiopathic, cholestasis, infection, and Wilson disease. In EEG results, normal, epileptic, diffuse slowing, and voltage suppression were 30%, 24%, 36%, and 9%, respectively. It was determined that all patients received liver support treatment, 22 patients (66.7%) underwent plasmapheresis, and 19 patients (57.6%) underwent liver transplantation (Table 1).

**Figure 3:**
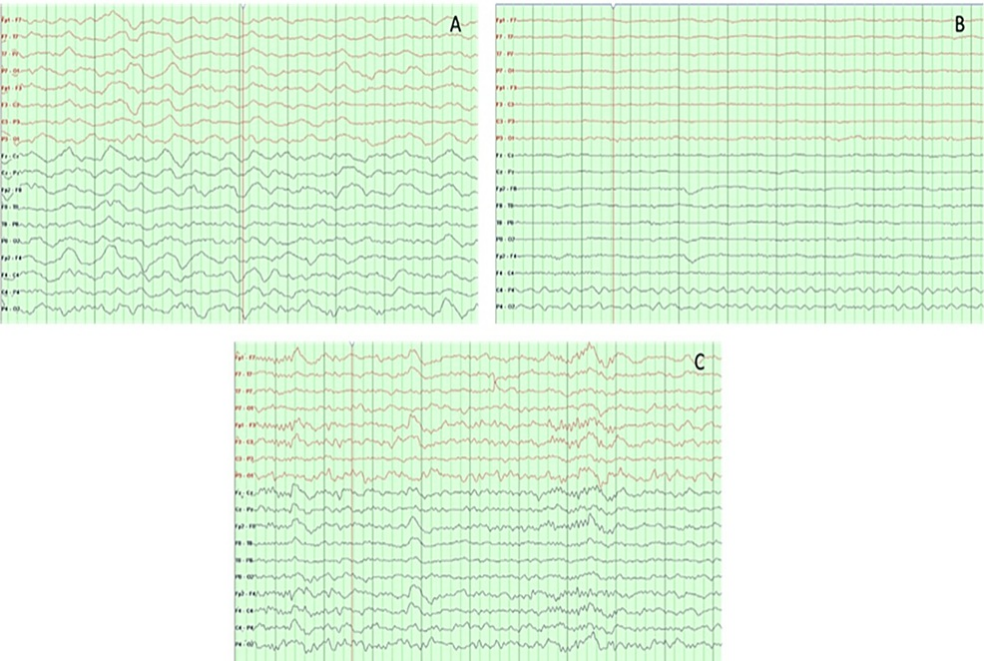
Patients' EEG finding samples: (A) diffuse slowing pattern, (B) voltage suppression pattern, and (C) normal EEG EEG: electroencephalogram

When the patients were evaluated according to the EEG findings, epileptic changes were present in eight patients (24%), diffuse slowing in 12 patients (36%), and voltage suppression in three patients (9%). Ten patients (30%) had normal EEG findings (Figure 3).

Based on the Child-Pugh scoring system, a new scoring system (TAI) consisting of only numerical parameters was created. EEG findings were added instead of encephalopathy. When the TAI score differences were evaluated according to the EEG findings of the patients, the Child-Pugh and TAI scores of the group with encephalopathy were found to be significantly higher than those with epileptic and normal EEG findings (p=0.016 and p=0.002, respectively) (Table 2).

**Table 2:** Best cut-off point for encephalopathy in Child-Pugh and TAI scores Logistic regression analysis showed that a TAI score of ≥7.5 increases the risk of developing encephalopathy 14.4 times (p=0.004; 95% CI: 2.289-90.597) Statistical method: ROC curve analysis CI: confidence interval; TAI: total bilirubin, albumin, and international normalized ratio; ROC: receiver operating characteristic

	Best cut-off point for encephalopathy	Sensitivity	Specificity	95% CI	P-value
Child-Pugh score	≥10.5	73.9	100	0.726-0.996	<0.001
TAI score	≥7.5	78.3	80	0.687-0.974	0.003

**Figure 4:**
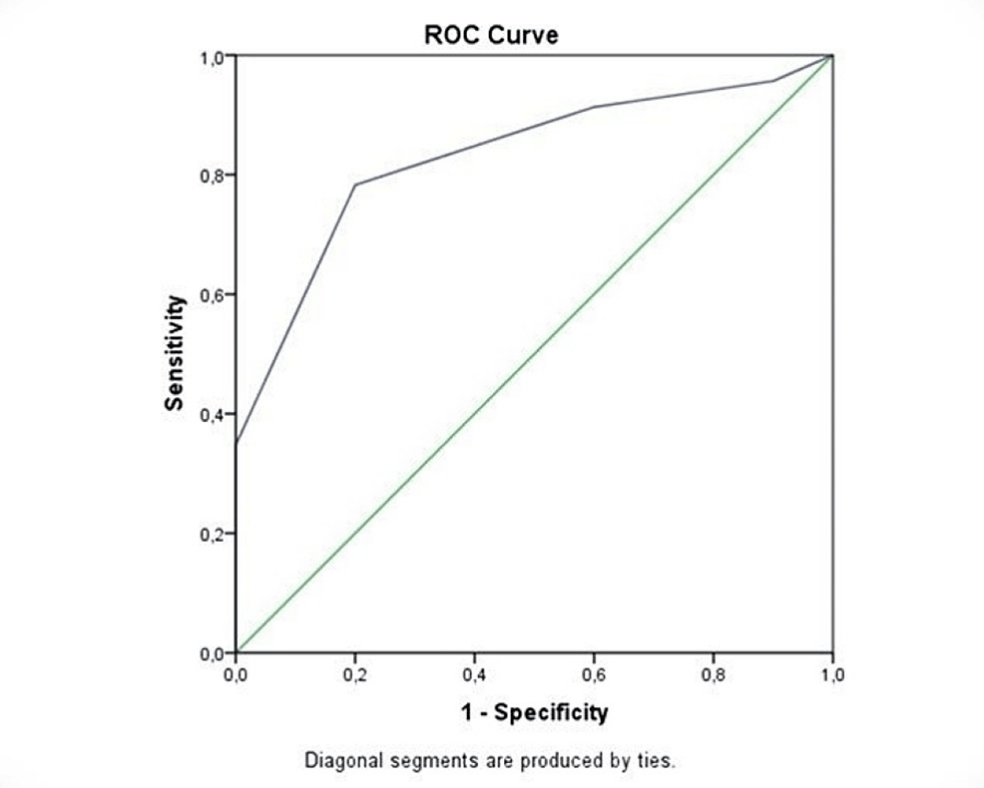
Best cut-off point for encephalopathy for TAI score in ROC curve analysis TAI: total bilirubin, albumin, and international normalized ratio; ROC: receiver operating characteristic

ROC curve analysis was performed to determine the best Child-Pugh and TAI score cut-off point to predict the presence of encephalopathy. Accordingly, if the Child-Pugh score is ≥10.5, it predicts the presence of encephalopathy with 73.9% sensitivity and 100% specificity (Table 3). However, since the presence of encephalopathy in the Child-Pugh score was also included in the scoring, we excluded it from the scoring and re-analyzed the ROC curve according to the TAI scoring values (Figure 4). Using the ROC curve analysis, the best cut-off point for the TAI score to detect encephalopathy was calculated. In agreement, if the TAI score is ≥7.5, we showed that it can detect encephalopathy with 78.3% sensitivity and 80% specificity.

**Table 3:** Evaluation of EEG findings and Child-Pugh and TAI scores Statistical method: one-way ANOVA EEG: electroencephalogram; TAI: total bilirubin, albumin, and international normalized ratio; ANOVA: analysis of variance

EEG findings	Normal	Epileptic	Encephalopathy		P-value
Child-Pugh score	9.80±1.68	9.50±1.41	11.47±1.80	0.016	
TAI score	7.00±1.05	7.00±1.31	8.33±0.72	0.002	

Logistic regression analysis showed that a TAI score of ≥7.5 increases the risk of developing encephalopathy 14.4 times (p=0.004; 95% CI: 2.289-90.597). EEG findings and treatment methods were compared. According to this comparison, it was determined that 9.643 times more plasmapheresis was performed in patients with diffuse slowing of EEG (p=0.012; 95% CI: 1.643-56.925).

When the rates of plasmapheresis and liver transplantation were evaluated according to the EEG findings of the patients, we found that plasmapheresis was performed significantly more in patients with diffuse slowing in EEG (p=0.007) and liver transplantation was performed significantly less in the group with normal EEG findings (p=0.005). Our study showed that patients with acute liver failure who required intensive care and had normal EEG findings could be discharged with liver support therapy alone, without the need for liver transplantation (p=0.005). We found that there was no significant difference in the patient groups with epileptic EEG compared to the group that did not undergo plasmapheresis and/or liver transplantation (p=0.350 and p=0.185, respectively). It was observed that there was no statistical difference in the patient groups with epileptic EEG compared to the group that did not undergo plasmapheresis and/or liver transplantation (p=0.706 and p=0.744, respectively) (Table 4).

**Table 4:** Evaluation of EEG findings and treatment Statistical methods: cross-tabs, chi-squared test, p<0.005 EEG: electroencephalogram

EEG findings	Plasmapheresis n-%	P-value	Liver transplantation n-%	P-value
Normal	5-22.7	0.540	1-5.9	0.005
Epileptic	2-9.1	0.350	2-11.8	0.185
Diffuse slowing	15-68.2	0.007	11-57.9	0.393
Voltage suppression	3-13.6	0.706	2-50	0.744

## Discussion

In childhood acute liver failure, EEG has been used since 1955 to evaluate the differential diagnosis of encephalopathy (e.g., nonconvulsive status, focal lesions). There is very limited information on whether EEG is of a prognostic value and/or a parameter to be included in the management of PELD/MELD or Child-Pugh scoring methods [16,17]. Altered consciousness in acute liver failure is seen as a wide spectrum from mood changes to deep coma. Although a multitude of possible pathophysiological hypotheses have been proposed for its occurrence, the effective mechanisms are not clear. The two main known mechanisms (together or separately) are hyperammonaemia, cytokines, neurotransmission imbalances and receptor alterations, metabolic encephalopathy, and brain edema [17,18]. While there are invasive and non-invasive methods used for the diagnosis of brain edema, detailed neurological examination and EEG results are used for the diagnosis of encephalopathy [19]. EEG is performed after the neurological examination in the evaluation of encephalopathy. Abnormal changes can be detected in the EEG before the clinical signs of encephalopathy appear, especially in children [20]. Encephalopathy is an important finding in the progression of PALF and may be undiagnosed with neurological examination in children [21]. EEG is a valuable diagnostic technique in the identification of encephalopathies. In addition, EEG abnormalities in children occur earlier than in adults [22]. In adult studies, the EEG findings of encephalopathy have been staged as alpha coma, triphasic wave, and periodic patterns; however, these abnormal morphologies were not common in children. On the other hand, the pediatric EEG findings include varying degrees of slowing, unresponsiveness to external stimuli, and discontinued activity [22,23]. In our study, we classified encephalopathic EEG findings into three stages, normal, diffuse slowing, and voltage suppression, which is different from the adult EEG abnormalities. Clinically, encephalopathy may reflect diffuse slowing and/or voltage suppression on the EEG. In our study, clinical encephalopathy was found in 70% of the patients, and encephalopathy was defined by EEG in 45% of the patients. Our study shows that EEG has an important role to play in diagnosing childhood encephalopathy. We sought an answer to the question of whether EEG findings can guide treatment selection, management, and prognostic considerations in this study.

Hussain and Nordli reported that the rate of liver transplantation or mortality would increase in patients with moderate or severe background rhythm abnormality in EEG. However, they did not specify the type of background rhythm abnormality. Furthermore, EEG findings and treatment options were not evaluated as we did, and only prognosis was emphasized [24]. The PALF study of 33 patients and 70 controls found a correlation between EEG and the severity of PALF, but the study emphasized the reliability of spectral EEG in this regard [25]. Our study demonstrated that patients with normal EEG findings less commonly underwent liver transplantation or liver support therapy as the previous series reported in the literature [26]. We found that patients with diffuse slowing in EEG were significantly more likely (9.643 times) to undergo plasmapheresis in our study. In the literature, we did not recognize any study that compared between plasmapheresis and EEG relation. When diffuse slowing was detected, plasmapheresis was considered the primary treatment, and liver transplantation was the next-line treatment. However, in our series, the same recommendation was not valid for cases with voltage suppression in EEG.

Although seizure is the most common neurological complication before and after liver transplantation in children, the seizure incidence is not known. Seizures are the most common complication after liver transplantation, occurring with a frequency of 2.8-42% [27]. Previously, the seizure incidence was 11.5% after liver transplantation in our department [27]. In a study of adults and children examining the role of EEG in HE, the incidence of seizures was found to be 20.1% [16]. The seizure incidence was found to be 24.2% in this study as similar. In all patients with seizures, EEG findings showed an epileptic abnormality. We did not find any effect of epileptic abnormality in EEG for liver failure treatment management. However, this result may be due to the small size of our case series.

The relationship between EEG findings and the prognosis of children with acute liver failure is an intriguing issue. In a retrospective study, the relationship between EEG findings and prognosis in 151 children and adults with acute liver failure was evaluated [16]. While abnormal brain imaging did not affect prognosis, it was found to be associated with EEG. It has been reported that the presence of slight EEG changes increases a patient's chance of being discharged. Another retrospective study including solely pediatric cases evaluated 19 patients requiring intensive care treatment; they reported that either transplantation was required or the patients died when they had moderate and severe background rhythm abnormalities in EEG [26]. Our study showed that patients with acute liver failure who needed intensive care and whose EEG findings were normal could be discharged with only liver support therapy, without liver transplantation being needed (p=0.005).

The Child-Pugh prognostic scoring criteria used in liver failure include clinical findings, such as encephalopathy, ascites, and liver disease, as well as numerical laboratory parameters, such as INR, albumin, and total bilirubin. These laboratory parameters are also available in the PELD mortality score. The PELD score accurately predicts the probability of death in the three-month waiting list for children with chronic liver disease [11]. Clinically, detecting encephalopathy in children is more challenging than in adults. EEG can be a very valuable diagnostic tool if appropriate imaging and expert interpreters are available, but it is sometimes not accessible. We have tried to find an answer to the question of whether the presence of encephalopathy in a patient with PALF might be predicted from laboratory parameters without EEG. We could not find any study in the literature relating laboratory values (total bilirubin, albumin, and INR) to encephalopathy. Therefore, in our study, we developed the TAI score, hypothesizing whether we could predict encephalopathy by using numerical laboratory parameters (total bilirubin, albumin, and INR) already included in the Child-Pugh score. Bilirubin, albumin, and INR values as well as the rate of the new encephalopathic EEG findings were found to be significantly higher than those in patients with normal and epileptic EEG. We compared our score with the Child-Pugh score, which demonstrated encephalopathy the best, and determined a cut-off value for our score. In our newly created scoring system, we determined that a total score (the cut-off value determined by the ROC curve analysis) of greater than 7.5 detected encephalopathy with a sensitivity of 78.3% and a specificity of 80% (p=0.003). Another important point that deserves mentioning about the TAI score is that it could indicate the risk of encephalopathy. The logistic regression analysis showed that a TAI score of ≥7.5 increased the risk of developing encephalopathy by 14.4 times in our study.

Limitations

The limitations of our study include its retrospective nature, small sample size, and no control clinical and laboratory evaluation. If we had performed continued EEG, we should have more information and timing of encephalopathy. Also, electrographic seizures and nonconvulsive status could be detected. Therefore, the importance of the TAI score against other scores should be established with statistical studies in the larger population. However, prospective, multicenter, randomized controlled studies with larger series are seriously needed in this field.

## Conclusions

In patients with a normal EEG, the requirement for liver transplantation is quite low, and the prognosis is more favorable. We noticed that plasmapheresis was performed 9,643 times more often in patients with diffuse slowing. Thus, plasmapheresis should be prioritized for treatment if diffuse slowing is detected on the EEG. EEG findings in children with acute hepatic failure show that they can be used as a tool for treatment selection and prediction of prognosis. The presence and risk of developing encephalopathy can be predicted using TAI scoring in patients with PALF especially challenging to diagnose encephalopathy. Clinicians should be guided by these findings. However, prospective, multicenter, randomized controlled studies with larger series are seriously needed in this field.
